# Adolescent Sjogren’s syndrome presenting as psychosis: a case series

**DOI:** 10.1186/s12969-020-0412-8

**Published:** 2020-02-11

**Authors:** Erin K. Hammett, Cristina Fernandez-Carbonell, Courtney Crayne, Alexis Boneparth, Randy Q. Cron, Suhas M. Radhakrishna

**Affiliations:** 1 Department of Pediatrics, Division of Rheumatology at Rady Children’s Hospital, 3020 Childrens Way, San Diego, Ca 92123 USA; 2 Department of Pediatrics, Division of Neurology at Children’s Hospital at Montefiore, 3415 Bainbridge Ave, Bronx, NY 10467 USA; 3 Department of Pediatrics, Division of Rheumatology at Children’s of Alabama, University of Alabama at Birmingham, 1600 7th Ave S, CPPN, Suite G10, Birmingham, AL 35233-1711 USA; 4 Department of Pediatrics, Division of Allergy, Immunology, and Rheumatology at Columbia University Medical Center, 630 W 168th St, New York, NY 10032 USA

**Keywords:** Central nervous system (CNS) Sjogren’s syndrome, Pediatric Sjogren’s syndrome, Psychosis

## Abstract

**Background:**

Neurological involvement has been reported in up to 80% of adults with Primary Sjogren’s syndrome (pSS) with psychiatric abnormalities including anxiety, depression, and cognitive dysfunction being common. Psychosis due to pSS has been reported in adult patients but has never been previously reported in the adolescent/pediatric literature. Here we describe for the first time four cases of adolescent Sjogren’s syndrome that presented with psychotic symptoms. Rituximab treatment was followed by improvement of psychiatric symptoms in all patients.

**Case presentation:**

**1**: 16 year old female without significant past medical history presented to the emergency department with 4 days of abnormal behavior, tremors, insomnia, polyphagia, polyuria, and suicidal ideation.

**2**: 16 year old female with a 4 year history of severe anxiety, OCD, and tic disorder treated with fluoxetine with partial benefit presented with an abrupt and severe worsening of anxiety, OCD and new auditory hallucinations.

**3**: 19 year old female without significant past medical history presented with a 3 day history of progressively altered behavior, incoherent speech, insomnia, headache, and tangential thoughts.

**4**: 17 year old female without significant past medical history presented with new onset suicidal ideation, paranoia, confusion, and emotional lability.

**Conclusion:**

Psychosis is more common in autoimmune disease than previously known. To our knowledge, the four teenage women described above are the first reported patients with adolescent pSS manifesting as psychosis. pSS should be considered in the differential diagnosis of young patients with new psychiatric disorders, even in the absence of sicca symptoms. Psychiatric symptoms improved with rituximab infusions in all 4 of our patients, which suggests rituximab may be an effective treatment option that should be considered early after the diagnosis of pSS-associated psychiatric disturbance.

## Introduction

Primary Sjogren’s syndrome (pSS) is a systemic autoimmune disease typically characterized by plasma-lymphocytic infiltration of the salivary and lacrimal glands. Dry mouth is a common presenting complaint in adults, while parotid swelling may be a more frequently encountered in children [1]. While inflammation is primarily directed toward the exocrine glands, extraglandular manifestations can include arthritis, Raynaud’s phenomenon, purpura, pulmonary disease, renal disease, and neurological involvement. Sjogren’s syndrome can also occur as a secondary condition with underlying systemic lupus erythematosus (SLE) or rheumatoid arthritis (RA). It most often affects middle-aged females with a prevalence of 0.1–3% and incidence of 3.9–5.3 yearly per 100,000 in the adult population [2]. No such epidemiologic data are available for children.

Neurological involvement has been reported in up to 80% of adults with pSS [3, 4] and can preceed the diagnosis in up to 50–80% of the cases [5, 6]. Central nervous system (CNS) manifestations include aphasia, dysarthria, visual loss, aseptic meningitis, transverse myelitis, neuromyelitis optica, and cognitive dysfunction [6]. Psychiatric abnormalities have also been described including depression, anxiety, and cognitive deficits [3]. Psychosis has been reported in adult patients [3, 7–12] with pSS but has never been previously reported in the pediatric literature, although other psychiatric manifestations including major depressive disorder and obsessive compulsive disorder (OCD) have been described in pediatric patients [13].

Brain magnetic resonance imaging (MRI) abnormalities, including non-enhancing T2 hyperintensities in the periventricular and subcortical areas, have been seen in up to 75% of adults with pSS and neuropsychiatric symptoms and 9% of adults with pSS without neuropsychiatric symptoms [14]. Abnormal electroencephalograms (EEG) are found in about 33–48% of patients with CNS pSS [15, 16]. Cerebrospinal fluid (CSF) analysis may be normal or show elevated IgG index [17]. Autopsy and brain biopsy studies in adults have also demonstrated inflammatory changes in pSS patients despite normal brain MRI and cerebral angiogram [18]. Biopsy findings in neurological involvement of Sjogren’s syndrome include small vessel vasculitis of the small cerebral blood vessels and direct inflammatory infiltration of cerebral tissue [15, 19]. There is also evidence for activation of the complement pathway [20]. Patients with anti-Sjogren’s syndrome type A (SSA) antibodies are more likely to have CNS disease than those with anti-Sjogren’s syndrome type B (SSB) [21]. Patients with HLA-DR3/DR4 may have greater risk of CNS disease while HLA-DR1, DR2, and DRw6 may be protective [22].

Treatment for CNS manifestations of Sjogren’s syndrome has been empiric, guided by expert opinion and anecdotal reports. Some experts recommend monthly pulse cyclophosphamide for 6–12 months in patients with progressive nervous system dysfunction [15]. Other immunosuppressive medications used with varying success include azathioprine, methotrexate, and cyclosporine [23]. Rituximab has been used anecdotally in CNS pSS with varying results [24, 25].

Here we describe for the first time four cases of adolescent Sjogren’s syndrome presenting with psychotic symptoms. Rituximab treatment was followed by improvement of psychiatric symptoms in all the patients.

## Case presentations

### Case 1

A 16 year old female without significant past medical history presented to the emergency department (ED) with 4 days of abnormal behavior, tremors, insomnia, polyphagia, polyuria, and suicidal ideation. She reported that she was sexually assaulted and had ingested synthetic cathinones (bath salts). Physical exam was within normal limits (WNL). Laboratory evaluation with complete blood count (CBC), complete metabolic panel (CMP), and thyroid studies were within normal limits. Head computed tomography (CT) showed no acute intracranial abnormality. She was discharged with a diagnosis of drug-induced psychiatric disturbance.

Over the course of the next few months the patient was seen in the ED six further times for suicidal ideation, drug use (methamphetamines and cocaine), and violence toward her family members. She had paranoid delusions that her family was trying to poison her and complained that “someone stole my nose.” She had mood lability and was noted to have abnormal hand gestures. She had cognitive deficits with inability to complete basic reading and writing assignments.

The patient was admitted to the hospital for further workup of psychosis, rheumatology was consulted for a recently discovered positive antinuclear antibody (ANA) of 1:1280 titer. She denied recent drug use in the last 6 months which her family confirmed. On physical exam she had tenderness to palpation of her parotid glands. Laboratory evaluation was positive for SSA > 8 (nl < 8) iu/ml. All other tests were within normal limits (Table 1): CBC, CMP, erythrocyte sedimentation rate (ESR), C reactive protein (CRP), urine analysis (UA), and urine drug screen were all normal. CSF cells, glucose and protein, C3 Complement (C3), C4 complement (C4) and thyroid stimulating hormone (TSH) were all also WNL. Antibodies to thyroid peroxidase (TPO), ANCA (antineutrophil cytoplasmic antibodies), double stranded deoxyribonucleic acid (dsDNA), ribosomal P, neuronal, N-methyl-D-aspartate receptor (NMDA), Smith, ribonucleoprotein (RNP), Beta 2 glycoprotein/cardiolipin, neuromyelitis optica (NMO)/aquaporin-4 (AQP4), and SSB were negative/WNL. Serum autoimmune encephalopathy panel (performed at Mayo lab) was negative. MRI of the brain showed symmetrical bilateral volume loss of the parietal lobes of uncertain significance. EEG was within normal limits (Table 2).

**Table 1:** Presenting Lab Results

Initial Labs	Case 1	Case 2	Case 3	Case 4
CBC/CMP	WNL	WNL	WNL	AST 62 U/L, bilirubin 1.11 U/L
SSA	> 8 iu/mL	4.8 AI	4.8 AI	8 iu/mL
SSB	Negative	> 8 AI	> 8 AI	Negative
CRP	WNL	WNL	1.1 mg/dL	WNL
ESR	WNL	57 mm/hr	60 mm/hr	WNL
C3/C4	WNL	WNL	WNL	WNL/9.6 mg/dL
ANA	1:1280 Speckled	1:1280 Speckled	1:160 mixed	1:640 speckled
dsDNA/Smith/RNP AB	Negative	Negative	Negative	Negative
Ribosomal P/Neuronal/NMDA AB	Negative	Negative	Negative	Negative
APS Panel	Negative	Negative	Negative	Negative
Encephalopathy Panel	Negative	Negative	Negative	Negative
TSH/TPO	WNL/Negative	WNL/Negative	WNL/Negative	WNL/Negative
Immunoglobulins	WNL	IgG 2116 mg/dL	WNL	WNL
RF	WNL	58.7 IU/mL	WNL	WNL
UA/Utox	WNL	WNL	WNL	WNL

Key:

*CBC* complete blood count, *CMP* complete metabolic panel, *AST* *Aspartate aminotransferase*, *SSA* Sjogren’s syndrome type A, *SSB* Sjogren’s syndrome type B, *CRP* C reactive protein, *ESR* erythrocyte sedimentation rate, *C3* C3 Complement, *C4* C4 Complement, *ANA* antinuclear antibody, *dsDNA* double stranded deoxyribonucleic acid, *RNP* ribonucleoprotein, *AB* antibody, *NMDA* neuronal, N-methyl-D-aspartate receptor, *APS* antiphospholipid syndrome, *TSH* thyroid stimulating hormone, *TPO* thyroid peroxidase, *RF* rheumatoid factor, *UA* urinalysis, *Utox* Urine toxicology

**Table 2:** Disease Summary by Case

	Case 1	Case 2	Case 3	Case 4
Age	16	16	19	17
Gender	Female	Female	Female	Female
Initial Presentation	Insomnia, polyphagia, polydipsia, SI, abnormal behavior	Anxiety, OCD, auditory hallucinations	Abnormal behavior, incoherent speech, insonia, HA, tangential thoughts	SI, paranoia, confusion, emotional lability, intermittent unresponsiveness, nocturnal enuresis
MRI Brain Findings	Bilateral volume loss of the parietal lobes	L frontal lobe focus of white matter change	WNL	WNL
CSF Findings	WNL, no pleiocytosis or increased oligoclonal bands	WNL, no pleiocytosis or increased oligoclonal bands	WNL, no pleiocytosis or increased oligoclonal bands	WNL, no pleiocytosis or increased oligoclonal bands
EEG Findings	WNL	Occasional delta range slowing in the left fronto-central-temporal region	WNL	WNL
Initial Immunosuppresive tx	Rituximab 1000 mg q 2 weeks × 2 and pulse methylprednisolone 1000 mg × 3 days followed by prednisone 60 mg tapered over 24 weeks	Rituximab 1000 mg × 1	Methylprednisolone 30 mg/kg IV and rituximab 1000 mg q 2 weeks × 2	Methylprednisolone (8 mg/kg/day IV) followed by oral prednisone (1.3 mg/kg/day) and IVIG 2 g/kg; then rituximab 1000 mg and pulse methylprednisolone; then IV plasmapharesis, rituximab 1000 mg and IV cyclophosphamide 750 mg/m2
Time to Improvement	2–5 months	1–5 months	2 months	1 week - 7 months
Length of Follow Up Since Initial tx	18 months	12 months	18 months then lost to follow-up	7 months
Total # rounds of anti-CD20 tx	1	2.5	2	2
Maintenance Therapy	None	Obinutuzumab q 6 months	Rituximab 1000 mg q2 weeks × 2 every 6 months, mycophenolate mofetil 1500 mg bid	Rituximab 1000 mg q2 weeks × 2 every 6 months, hydroxychloroquine 200 mg daily and prednisone 0.65 mg/kg/day
Currently Off Antipsychotics?	Yes	No	Yes	Yes

Key:

*OCD* Obsessive compulsive disorder, *WNL* Within normal limits, *Tx* Treatment, *SI* Suicidal Ideation, *HA* Headache

A minor salivary gland biopsy showed small foci of lymphoplasmacytic predominantly peri-ductal inflammatory infiltrate with > 50 infiltrates in three foci; giving her a focus score of 3. Schirmer’s test was abnormal at 5 mm bilaterally (normal > 10 mm) (Table 3). She was diagnosed with pSS based on the 2017 American College of Rheumatology (ACR)/European League Against Rheumatism classification criteria (EULAR) [26].

**Table 3:** 2016 ACR/EULAR Sjogren’s Classification Criteria Met

Sjogren’s Criteria	Case 1	Case 2	Case 3	Case 4
anti-SSa Antibody (3 points)	Y	Y	Y	Y
Focus score >1 (3 points)	Y	Not performed	Y	Not performed
Ocular Staining Score >5 or VBS >4 (1 point)	Not performed	Not performed	Not performed	Not performed
Schirmer’s test <5mm/ 5min (1 point)	Y	Not performed	Y	Not performed
Unstimulated Salivary Flow Rate <0.1mL/min (1 point)	Not performed	Not performed	Not performed	Not performed
Total score (Need 4 of 9)	7	3	7	3

*VBS* Van Bijsterveld score, *Y* Yes

The patient was initially treated with olanzapine on an inpatient psychiatry ward. After several months, her recovery was suboptimal and she continued to have severe cognitive deficits with difficulty in comprehension, reasoning, and memory suggesting a diagnosis other than a primary psychiatric disorder. After obtaining consent from her family, immunosuppressive treatment was initiated with 1000 mg rituximab every 2 weeks for two doses in addition to pulse dose methylprednisolone 1000 mg daily for 3 days followed by a prednisone taper over 24 weeks. Since initiation of immunosuppressive therapy the patient has been followed for 18 months and made major cognitive improvements, no longer has psychotic symptoms, and is off psychotropic medication. She has not developed any new symptoms or received further rituximab infusions.

### Case 2

A 16 year old female presented with a 4 year history of severe anxiety, OCD, and tic disorder treated with fluoxetine with partial benefit. Four months prior to evaluation, she developed an abrupt and severe worsening of anxiety, OCD and new auditory hallucinations and was started on aripiprazole which led to a reduction of her auditory hallucinations to approximately once per day. She was able to resume school on a modified schedule. Lab work up was notable for positive ANA 1:1280 (speckled), anti-SSA 4.8 (0.0–0.9) AI and anti-SSB > 8 (0.0–0.9) AI, elevated Immunoglobulin G (IgG)(2116) mg/dL, ESR 57 mm/hr., and positive rheumatoid factor (RF) (58.7 IU/mL). CBC, CMP, TSH and free thyroxine 4 (fT4), thyroid antibodies, UA, CSF analysis (including CSF autoimmune encephalitis antibody panel and oligoclonal bands) were within normal limits (Table 1). MRI brain revealed a single punctate focus of nonspecific white matter signal change in the left frontal lobe, and was otherwise unremarkable (Table 2). Magnetic Resonance spectroscopy brain imaging revealed abnormal brain perfusion with regional cerebral cortical left anterior temporal moderate hypoperfusion and relative minimal hypoperfusion in right thalamus of unclear significance. EEG showed occasional delta range slowing in the left fronto-central-temporal region. The patient did not consent to labial salivary gland biopsy and refused ophthalmology evaluation for Schirmer’s testing.

Despite the absence of sicca symptoms and not fulfilling the 2017 ACR/EULAR diagnostic criteria (Table 3), the patient’s positive serologies and evidence of organic brain disease on MRI spectroscopy and EEG prompted the presumptive diagnosis of neuropsychiatric pSS. The patient was treated with a single dose of rituximab 1000 mg. She then presented with fever, rash and joint pain 10 days after the rituximab infusion. She was found to have platelet count of 124 × 10^3/uL, elevated CRP of 91.4 mg/L, low C4 < 4 mg/dL, C3 of 138 mg/dL, with otherwise unremarkable CBC and CMP. She was diagnosed with rituximab-induced serum sickness reaction and was treated with a 1 week course of prednisolone with resolution of her serum sickness symptoms. Repeat labs showed normal platelet count of 370 and repeat C4 of 11 mg/dL. A lymphocyte panel showed complete B cell depletion with 0% CD19+ cells. Plans for a second dose of rituximab were cancelled.

One month after treatment with rituximab the patient’s mother reported that the patient’s mood had improved. Five months later, the patient was having less auditory hallucinations, occurring approximately once a month and associated with times of increased stress. She returned to school and extra-curricular activities. She also reported significantly improved sleep quality and mood. She was able to decrease her aripiprazole dose by 50%. The patient and her parents declined treatment with hydroxychloroquine. Six months after her initial rituximab treatment, repopulation of her peripheral CD19+ cell count and previous improvement of psychiatric symptoms with rituximab prompted treatment with obinutuzumab, a humanized anti-CD20 monoclonal antibody that did not confer the same risk of serum-sickness reaction. She tolerated obinutuzumab treatment well. About 5 months after initial treatment with obinutuzumab the patient developed an increase in hallucinations, tics, and anxiety after starting an oral contraceptive (OCP). She was re-treated with obinutuzumab infusion, her OCP was discontinued, and her flare of psychiatric symptoms resolved. She has been followed for 12 months since initiation of immunosuppressive therapy without development of new symptoms. She has not received any further treatment with obinutuzumab or other immunosuppressive therapies.

### Case **3**

A 19-year-old female with no significant past medical or psychiatric history presented with a three-day history of progressively altered behavior, incoherent speech, insomnia, headache, and tangential thoughts. In the months preceding initial presentation she was a victim of sexual assault and experienced the death of a close friend. She denied any use of drugs, tobacco or alcohol.

Physical exam was notable for temperature of 100.7 F. Mental status exam was remarkable for disorganized and repetitive speech, flight of ideas and tangential thoughts. Laboratory tests showed a CRP of 1.1 (0.0–0.8) mg/dL and ESR of 60 (0–20) mm/h. CBC, CMP, UA, CSF cells, glucose and protein, UA, urine toxicology screen, salicylate and acetaminophen level were all normal (Table 1).

Soon after hospital admission, she developed an episode of bilateral hand tremors with upper extremity rigidity, tachycardia and systolic hypertension without fever or elevated inflammatory markers that improved after adding diphenhydramine and lorazepam. A few days later, she had another transient event characterized by posturing, mood lability and generalized tremors. Neurological exam showed generalized hyperreflexia but no focal abnormalities. Due to concern for a primary psychiatric disorder she was discharged to an inpatient psychiatric facility where she received risperidone, benztropine and clonazepam to assist with sleep. She was treated with this regimen for about a month, during this time she developed worsening staring episodes concerning for seizures and was transferred back to the primary hospital for further medical work up that revealed a positive ANA 1:160 mixed pattern, with anti-SSA 4.8 (0.0–0.9) AI and anti-SSB > 8.0 (0.0–0.9) AI. Antibody testing for anti-dsDNA, Smith, ribosomal P, Jo-1, topoisomerase I (SCL-70), centromere, paraneoplastic and autoimmune encephalitis panels (including NMO and NMDA antibodies), and TPO auto-antibodies were negative. UA, C3 and C4 were normal. Blood, urine and CSF cultures showed no growth. Ceruloplasmin, porphyrins, angiotensin converting enzyme, human immunodeficiency virus (HIV) and syphilis testing were negative. An abdominal and pelvic ultrasound (US) were normal. Brain MRI with contrast and brain magnetic resonance angiogram (MRA) were WNL (Table 2). She underwent continuous EEG that showed no evidence of seizure activity. She continued to display disorganized thoughts with fluctuating coherence, persistent delusions, slow psychomotor responses, echolalia, echopraxia and posturing. Symptoms markedly improved after initiation of lorazepam, which prompted a diagnosis of catatonia.

She was re-started on risperidone and then switched to aripiprazole due to extrapyramidal side effects. She continued to have marked psychiatric symptoms including auditory and olfactory hallucinations, echolalia, paranoia, hyper-religiosity, derailment and thought blocking. Given ongoing symptoms, she was restarted on risperidone along with benztropine and clonazepam.

She was diagnosed with primary Sjogren’s syndrome based on positive ANA, anti-SSA and anti-SSB titers as well as an abnormal Schirmer’s test and a minor salivary gland biopsy focus score of 1–2 (Table 3). She was started on prednisone 45 mg/day with transient improvement, but developed subsequent worsening of her paranoia with more episodes of staring, intermittent posturing and fluctuating mood after 10 days. Symptoms improved with IV methylprednisolone 30 mg/kg/day for 3 days, restarting aripiprazole and increased lorazepam. IV rituximab was started as a steroid sparing agent. She received the first dose 1.5 months after disease onset. Two months later, her psychotic symptoms and catatonia improved considerably and she was weaned off lorazepam, aripiprazole, and steroids. Her symptoms improved with rituximab infusions 500 mg/m2 (two doses 2 weeks apart) every 4 months, mycophenolate mofetil 1500 mg twice a day, and oral prednisone 2.5 mg/day and she was able to return to nursing school and her extracurricular activities. Neuropsychological assessment 4 months after diagnosis revealed overall low average intellectual ability with weakness in nonverbal reasoning, visuospatial abilities, working memory, processing speed, attention, planning and organization. She received a total of 2 rounds of rituximab and was followed for 18 months after initiation of immunosuppressive medication without development of new symptoms but was subsequently lost to follow up.

### Case **4**

A previously healthy 17 year old female presented with new onset suicidal ideation, paranoia, confusion, and emotional lability. She had no auditory or visual hallucinations. Review of systems was otherwise negative. Her mother was noted to have a history of RA.

Physical exam was notable for flat affect but was otherwise normal. Within the first few days of presentation, her agitation worsened and she became intermittently unresponsive. She also had episodes of nocturnal enuresis. MRI brain with and without contrast was normal (Table 2). EEG was normal. All initial laboratory testing was normal apart from aspartate aminotransferase (AST) 62 (< 26) U/L, bilirubin 1.11 (nl < 0.84) mg/dL, and C4 9.6 (nl > 13) mg/dL. CBC, ESR, CRP, UA, CSF cell counts, glucose, and protein, rapid plasma reagin (RPR) and treponemal Ab, herpes simplex viruses (HSV) polymerase chain reaction (PCR) were normal / negative (Table 1). On day four of admission she received 3 days of pulse methylprednisolone (8 mg/kg/day IV) followed by oral prednisone (1.3 mg/kg/day), and on day 7 received IVIG (2 g/kg, max 100 g) without improvement in her psychotic state.

Following empiric treatment, further laboratory testing revealed positive ANA 1:640 in a speckled pattern, Anti-SSA 8 (nl < 1) iu/mL. Other antibody testing for ANCA panel, RF, ribosomal P Ab, Smith, RNP, SSB, anti-Scl70, and anti-dsDNA Ab were negative. She was started on ziprasidone and risperidone. Minor salivary gland biopsy/ keratoconjunctivitis sicca testing was not performed. Given persistent psychosis despite treatment with antipsychotics, positive SSA-antibody, and low C4 without further signs or symptoms of SLE, she was presumptively diagnosed with primary Sjogren’s syndrome (Table 3) and treated with rituximab 1000 mg IV and pulse methylprednisolone for 3 days followed by oral prednisone taper. Her mental status improved and she was discharged home on hydroxychloroquine 200 mg daily and prednisone 0.65 mg/kg/day.

Approximately 12 days after her first rituximab dose, the patient was readmitted for worsening suicidal ideation and new auditory hallucinations. Repeat CMP showed an increase in liver enzymes (AST 49, alanine aminotransferase (ALT) 106 mg/dL). The patient received the second dose of rituximab and one dose of IV cyclophosphamide 750 mg/m2 and was transitioned to oral prednisone. Her mental status improved over a period of weeks to months. She received a second round of rituximab 6 months later. She is currently being treated for depression/anxiety but was able to wean off all antipsychotics, and now has normal cognitive function. She has been followed for 7 months since initiation of immunosuppressive treatment without development of new symptoms.

## Discussion

Psychosis is defined as a condition that affects the mind where there has been some loss of contact with reality, often with deficits in cognitive processing commonly manifested as hallucinations or delusions [27]. It is separated into primary (idiopathic) and secondary causes due to medical illnesses or substance use [28]. Secondary psychosis is more common in autoimmune disease than previously recognized [29, 30]. It is a common manifestation of neuropsychiatric SLE and has been seen in about 12% of the pediatric neuropsychiatric SLE population [31]. Psychiatric disturbance has also been well documented in various case reports and case series in the adult pSS population [3, 4, 10, 16, 32, 33], including depressive disorder, anxiety disorder and sleep disorder [34]. The reported incidence of psychiatric and/or cognitive impairment in adult pSS patients has been under great debate varying from < 10% in a prospective study of pSS seen in a rheumatology clinic [16] to 80% in a retrospective study [11]. This variability is thought to be due to a lack of definition of CNS manifestations in pSS with inclusion of mild symptoms such as headache and cognitive dysfunction in some studies, use of primary versus secondary Sjogren’s syndrome population and differences in diagnostic criteria used to define Sjogren’s syndrome [35]. Frank psychosis in adult pSS has been seen less commonly and has not yet been documented in the pediatric or adolescent population.

To our knowledge, the four young women described above are the first reported patients with adolescent pSS manifesting as psychosis. None of the patents presented above had symptoms or lab criteria to suggest a diagnosis of SLE. A history of sexual assault was reported in cases 1 and 3 and drug use was reported in case 1. In both of these cases the psychiatric symptoms were initially attributed to primary psychiatric illness, with the diagnosis of psychosis related to pSS being made only after conventional psychiatric treatment had failed. Although one must consider the possibility that these patient’s psychiatric symptoms were related to substance abuse/traumatic life events, the fact that the patients did not respond typically to anti-psychotic medication and the positive response seen with immunosuppressive medication suggests the psychotic symptoms were indeed related to their confirmed diagnosis of pSS.

In cases 2 and 4 the diagnosis of pSS was based on the presence of positive anti-SSA serology alone as keratoconjunctivitis sicca testing and minor salivary gland biopsy were not performed; therefore these patients did not satisfy the 2017 ACR-EULAR criteria for pSS [26]. It is important to remember that there are no diagnostic criteria for pSS in the pediatric patient population, and childhood presentations can be quite variable often with extra-glandular manifestations predominating [36]. None of the 4 patients presented above complained of keratoconjunctivitis sicca or xerostomia. It is impossible to exclude the possibility that these patients had primary psychiatric disease and not secondary psychosis related to pSS. However, given the suspicion for organic brain disease in these patients as well as the known association of psychosis with pSS, the risk benefit analysis favored empiric treatment for presumed pSS and all patients showed improvement following treatment with rituximab. Three of the four patients have been weaned off anti-psychotics.

## Conclusion

Our case series suggests that psychiatric symptoms, mainly psychosis, can be an initial presentation of pSS in the adolescent population. Thus, pSS should be considered in the differential diagnosis of young patients with new psychiatric disorders, even in the absence of sicca symptoms. Psychiatric symptoms improved with rituximab/anti-CD20 infusions in all 4 of our patients, which suggests that rituximab may be an effective treatment option that should be considered early after the diagnosis of pSS-associated psychiatric disturbance.

## Data Availability

Data sharing is not applicable to this article as no datasets were generated or analyzed during the current study.

## Abbreviations

ACR: American College of Rheumatology
ALT: Alanine aminotransferase
ANA: Antinuclear antibody
ANCA: Antineutrophil cytoplasmic antibodies
AQP4: Aquaporin-4
AST: Aspartate aminotransferase
C3: C3 Complement
C4: C4 Complement
CBC: Complete blood count
CMP: Complete metabolic panel
CNS: Central nervous system
CRP: C reactive protein
CSF: Cerebrospinal fluid
CT: Computed tomography
dsDNA: Double stranded deoxyribonucleic acid
ED: Emergency department
EEG: Electroencephalogram
ESR: Erythrocyte sedimentation rate
EULAR: European League Against Rheumatism classification criteria
fT4: Thyroxine 4
HIV: Human immunodeficiency virus
HSV: Herpes simplex viruses
IgG: Immunoglobulin G
MRA: Magnetic resonance angiogram
MRI: Magnetic resonance imaging
NMDA: Neuronal, N-methyl-D-aspartate receptor
NMO: Neuromyelitis optica
OCD: Obsessive compulsive disorder
OCP: Oral contraceptive
PCR: Polymerase chain reaction
pSS: Primary Sjogren’s syndrome
RA: Rheumatoid arthritis
RF: Rheumatoid factor
RNP: Ribonucleoprotein
RPR: Rapid plasma reagin
SCL-70: Topoisomerase I
SLE: Systemic lupus erythematosus
SSA: Sjogren’s syndrome type A
SSB: Sjogren’s syndrome type B
TPO: Thyroid peroxidase
TSH: Thyroid stimulating hormone
UA: Urinalysis
US: Ultrasound
WNL: Within normal limits

## References

[1] Virdee S, Greenan-Barrett J, Ciurtin C (2017). A systematic review of primary Sjögren's syndrome in male and paediatric populations. Clin Rheumatol.

[2] Tucker L. Sjogren’s Syndrome. In: Petty RE, Laxer RM, Lindsley CB, Wedderburn L, editors. Textbook of pediatric rheumatology. 7th ed: Philadelphia: Saunders; 2016. p. 427–35.

[3] Cox PD, Hales RE (1999). CNS Sjögren's syndrome: an underrecognized and underappreciated neuropsychiatric disorder. J Neuropsychiatry Clin Neurosci.

[4] Mauch E, Völk C, Kratzsch G, Krapf H, Kornhuber HH, Laufen H (1994). Neurological and neuropsychiatric dysfunction in primary Sjögren's syndrome. Acta Neurol Scand.

[5] Delalande S, de Seze J, Fauchais AL, Hachulla E, Stojkovic T, Ferriby D (2004). Neurologic manifestations in primary Sjögren syndrome: a study of 82 patients. Medicine (Baltimore).

[6] Massara A, Bonazza S, Castellino G, Caniatti L, Trotta F, Borrelli M (2010). Central nervous system involvement in Sjögren's syndrome: unusual, but not unremarkable--clinical, serological characteristics and outcomes in a large cohort of Italian patients. Rheumatology (Oxford).

[7] Ampelas JF, Wattiaux MJ, VanAmarongen AP (2001). Troubles psychiatriques du lupus erythmateux dissemine et du syndrome de Gougerot-Sjogren. L’ Encephale.

[8] Lin CE (2016). One patient with Sjogren's syndrome presenting schizophrenia-like symptoms. Neuropsychiatr Dis Treat.

[9] Rosado SN, Silveira V, Reis AI, Gordinho A, Noronha C (2018). Catatonia and psychosis as manifestations of primary Sjögren's syndrome. Eur J Case Rep Intern Med.

[10] Hietaharju A, Yli-Kerttula U, Häkkinen V, Frey H (1990). Nervous system manifestations in Sjögren's syndrome. Acta Neurol Scand.

[11] Spezialetti R, Bluestein HG, Peter JB, Alexander EL (1993). Neuropsychiatric disease in Sjögren's syndrome: anti-ribosomal P and anti-neuronal antibodies. Am J Med.

[12] Kang JH, Lin HC (2010). Comorbidities in patients with primary Sjogren's syndrome: a registry-based case-control study. J Rheumatol.

[13] Ong LTC, Galambos G, Brown DA (2017). Primary Sjogren's syndrome associated with treatment-resistant obsessive-compulsive disorder. Front Psychiatry.

[14] Alexander EL, Beall SS, Gordon B, Selnes OA, Yannakakis GD, Patronas N (1988). Magnetic resonance imaging of cerebral lesions in patients with the Sjögren syndrome. Ann Intern Med.

[15] Alexander EL (1993). Neurologic disease in Sjögren's syndrome: mononuclear inflammatory vasculopathy affecting central/peripheral nervous system and muscle. A clinical review and update of immunopathogenesis. Rheum Dis Clin N Am.

[16] Moll JW, Markusse HM, Pijnenburg JJ, Vecht CJ, Henzen-Logmans SC (1993). Antineuronal antibodies in patients with neurologic complications of primary Sjögren's syndrome. Neurology..

[17] Alexander EL, Lijewski JE, Jerdan MS, Alexander GE (1986). Evidence of an immunopathogenic basis for central nervous system disease in primary Sjögren's syndrome. Arthritis Rheum.

[18] Alexander E (1992). Central nervous system disease in Sjögren's syndrome. New insights into immunopathogenesis. Rheum Dis Clin N Am.

[19] Ferreiro JE, Robalino BD, Saldana MJ (1987). Primary Sjögren's syndrome with diffuse cerebral vasculitis and lymphocytic interstitial pneumonitis. Am J Med.

[20] Sanders ME, Alexander EL, Koski CL, Frank MM, Joiner KA (1987). Detection of activated terminal complement (C5b-9) in cerebrospinal fluid from patients with central nervous system involvement of primary Sjogren's syndrome or systemic lupus erythematosus. J Immunol.

[21] Alexander EL, Ranzenbach MR, Kumar AJ, Kozachuk WE, Rosenbaum AE, Patronas N (1994). Anti-Ro (SS-A) autoantibodies in central nervous system disease associated with Sjögren's syndrome (CNS-SS): clinical, neuroimaging, and angiographic correlates. Neurology..

[22] Jacob CO, Plitt JR, Fronek Z (1992). HLA class II associated tumor necrosis factor alpha synthesis in central nervous system disease in Sjogren’s syndrome. Arthritis Rheum.

[23] Vivino FB, Carsons SE, Foulks G, Daniels TE, Parke A, Brennan MT (2016). New treatment guidelines for Sjögren's disease. Rheum Dis Clin N Am.

[24] Mekinian A, Ravaud P, Larroche C, Hachulla E, Gombert B, Blanchard-Delaunay C (2012). Rituximab in central nervous system manifestations of patients with primary Sjögren's syndrome: results from the AIR registry. Clin Exp Rheumatol.

[25] Tokunaga M, Saito K, Kawabata D, Imura Y, Fujii T, Nakayamada S (2007). Efficacy of rituximab (anti-CD20) for refractory systemic lupus erythematosus involving the central nervous system. Ann Rheum Dis.

[26] Shiboski CH, Shiboski SC (2016). 2016 American College of Rheumatology/European league against rheumatism classification criteria for primary Sjögren’s syndrome: a consensus and data-driven methodology involving three international patient cohorts. Arthritis and Rheumatology.

[27] Arciniegas DB. Psychosis. Continuum (Minneapolis, Minn). 2015; 21(3 Behavioral neurology and neuropsychiatry): 713–736. 10.1212/01.CON.0000466662.89908.e7 PMC4455840 26039850

[28] Keshavan MS, Kaneko Y (2013). Secondary psychoses: an update. World Psychiatry.

[29] Chen SJ, Chao YL, Chen CY, Chang CM, Wu EC, Wu CS (2012). Prevalence of autoimmune diseases in in-patients with schizophrenia: nationwide population-based study. Br J Psychiatry.

[30] Eaton WW, Byrne M, Ewald H, Mors O, Chen CY, Agerbo E (2006). Association of schizophrenia and autoimmune diseases: linkage of Danish national registers. Am J Psychiatry.

[31] Sibbitt WL, Brandt JR, Johnson CR, Maldonado ME, Patel SR, Ford CC (2002). The incidence and prevalence of neuropsychiatric syndromes in pediatric onset systemic lupus erythematosus. J Rheumatol.

[32] Pelizza L, Bonacini F, Ferrari A (2010). Psychiatric disorder as clinical presentation of primary Sjögren's syndrome: two case reports. Ann General Psychiatry.

[33] Malinow KL, Molina R, Gordon B, Selnes OA, Provost TT, Alexander EL (1985). Neuropsychiatric dysfunction in primary Sjögren's syndrome. Ann Intern Med.

[34] Shen CC, Yang AC, Kuo BI, Tsai SJ (2015). Risk of psychiatric disorders following primary Sjögren syndrome: a Nationwide population-based retrospective cohort study. J Rheumatol.

[35] Soliotis FC, Mavragani CP, Moutsopoulos HM (2004). Central nervous system involvement in Sjogren's syndrome. Ann Rheum Dis.

[36] Lieberman SM (2013). Childhood Sjogren syndrome: insights from adults and animal models. Curr Opin Rheumatol.

